# The Polyclinic Dinner

**Published:** 1909-05-08

**Authors:** 


### THE POLYCLINIC DINNER.

The Ninth Annual Dinner of the Medical Graduates' College and Polyclinic will be held at the Trocadero Restaurant, Piccadilly Circus, on Monday, May 24, at 7.15 for 7.30 p.m., under the chairmanship of Professor Howard Marsh, M.A., M.C., F.R.C.S., Professor of Surgery in the University of Cambridge. During the evening a presentation will be made to Captain Pinch, F.R.C.S., late Medical Superintendent of the Polyclinic. The price of the dinner will be 7s. 6d. (exclusive of wine).

